# In Search for Titanocene Complexes with Improved Cytotoxic Activity: Synthesis, X-Ray Structure, and Spectroscopic Study of Bis(*η* ^5^-cyclopentadienyl)difluorotitanium(IV)

**DOI:** 10.1155/2010/914580

**Published:** 2010-06-23

**Authors:** Elias Koleros, Theocharis C. Stamatatos, Vassilis Psycharis, Catherine P. Raptopoulou, Spyros P. Perlepes, Nikolaos Klouras

**Affiliations:** ^1^Department of Chemistry, University of Patras, 26504 Patras, Greece; ^2^National Center of Scientific Research, “Demokritos” (NCSR), Institute of Materials Science, Aghia Paraskevi, 15310 Attikis, Greece

## Abstract

The 1 : 2 reaction of [Ti(*η* ^5^-C_5_H_5_)_2_Cl_2_] and AgF in CHCl_3_/H_2_O yielded the fluoro analog [Ti(*η* ^5^-C_5_H_5_)_2_F_2_] (**1**) in almost quantitative yield (C_5_H_5_ is the cyclopentadienyl group). The coordination about the Ti^IV^ atom formed by two fluoro ligands and the centroids of the cyclopentadienyl rings is distorted tetrahedral. The compound crystallizes in the orthorhombic space group *C2cm*. The lattice constants are *a* = 5.9055(4), *b* = 10.3021(5), *c* = 14.2619(9) Å, and *α* = *β* = *γ* = 90°. The complex has been characterized by elemental analyses and spectroscopic (IR, ^1^H NMR) data. A structural comparison of the four members of the [Ti(*η* ^5^-C_5_H_5_)_2_X_2_] family of complexes (X = F, Cl, Br, I) is attempted.

## 1. Introduction

One of the first metal complexes discovered to exhibit biological activity has been cisplatin, [Pt(NH_3_)_2_Cl_2_] [1]. It is considered as one of the most efficient drugs for the treatment of certain types of cancer; however, drug toxicity and resistance limit its utilization for a broader range of diseases. In recent years, there has been a growing interest in the development of nonplatinum-based anticancer therapeutics. The main goal is to increase the variety of potential drugs, which may lead to higher activities enabling the administration of lower doses, attack of different types of tumour cells, solution of drug resistance problems, better selectivity and to lower toxicity. Non-platinum complexes may introduce numerous options for coordination numbers and geometries, oxidation states, affinity for certain types of biological ligands, and so forth, and may thus operate by different mechanisms. One class of such complexes are metallocene dihalides.

Metallocene dihalides, [M(*η* ^5^-C_5_H_5_)_2_X_2_] (M = Ti, V, Nb, Mo, Re; X = halide ligand; C_5_H_5_ = Cp, the cyclopentadienyl group), are a relatively new class of small hydrophobic organometallic anticancer agents that exhibit antitumour activities against cancer cell lines, such as leukaemias P388 and L1210, colon 38 and Lewis lung carcinomas, B16 melanoma, solid and fluid Ehrlich ascites tumours and also against human colon, renal and lung carcinomas transplanted into athymic mice [2–5]. Titanocene dichloride, [Ti(*η* ^5^-C_5_H_5_)_2_Cl_2_], is the most widely studied metallocene compound as a cytotoxic anticancer agent, which means that it can selectively kill cancer cells, and was used in phase I and II clinical trials [6–13]. However, the efficacy of [Ti(*η* ^5^-C_5_H_5_)_2_Cl_2_] in phase II clinical trials in patients with metastatic renal cell carcinoma [12] or metastatic breast cancer [13] was too low to be pursued. As titanium is present in many biomaterials, such as in food in the form of a whitening pigment, it is not unreasonable to conceive that it may be incorporated into drugs and into living systems, with particularly low toxicity [14].

In 2008, a novel class of substituted titanocene dichlorides, the so-called “benzyl-substituted titanocenes”, with improved cytotoxic activity were developed and tested for their potential application as anticancer drugs [15]. The cytotoxic activity can also be influenced by substitution of the two chloride ligands. More recently, Huhn and co-workers reported the synthesis and cytotoxicity of selected benzyl-substituted fluorotitanocene derivatives that showed a cytotoxic activity 3–5 fold higher than that of the respective dichlorides [16]. In the same paper, the X-ray structure analysis of two of the titanocene difluoride derivatives was also described. The prototype of the latter compounds is titanocene difluoride, [Ti(*η* ^5^-C_5_H_5_)_2_F_2_], which itself (i) exhibits strong antitumor, anti-inflammatory and anti-arthritic activity as well as immunosuppressant effects [17, 18], (ii) reduces significantly the rates of crystal growth of hydroxyapatite (the model compound for the inorganic component of bones and teeth, observed in pathological calcifications of the articular cartilage) [19], and (iii) is an effective catalyst for the reduction of lactones and imines, reductive-deoxygenative coupling of amides, hydrogenation of olefins, and defluorination of saturated perfluorocarbons [20].

Surprisingly, the crystal structure of bis(*η* ^5^-cyclopentadienyl)difluorotitanium(IV) is not known. In the present work, we describe the first X-ray diffraction study of [Ti(*η* ^5^-C_5_H_5_)_2_F_2_], providing structural data which will probably be important in structure-activity investigations of new titanocene difluorides, a promising class in terms of medical applications.

## 2. Experiments

Reagents and solvents were purchased from commercial sources, and were purified (where necessary) and dried before use by standard procedures. The starting titanocene dichloride, [Ti(*η* ^5^-C_5_H_5_)_2_Cl_2_], was synthesized under an argon atmosphere using dried THF by the method of Wilkinson and Birmingham [21] and recrystallized from boiling toluene. All manipulations were performed under aerobic conditions. Microanalyses (C, H) were performed by the University of Patras (Greece) Microanalytical Laboratory using an EA 1108 Carlo Erba analyzer. IR spectra (4000–450 cm^−1^) were recorded on a Perkin-Elmer 16 PC FT-spectrometer with samples prepared as KBr pellets. The ^1^H NMR spectrum of the complex in CDCl_3_ was recorded with a Bruker Avance 400 MHz spectrometer; chemical shifts are reported relative to tetramethylsilane. Conductivity measurements were carried out at 25°C using an Ehrhardt-Metzger, type L21, conductivity bridge.

[Ti(*η* ^5^-C_5_H_5_)_2_F_2_] (**1**) was synthesized in a plastic bottle from a solution of the dichloride, [Ti(*η* ^5^-C_5_H_5_)_2_Cl_2_] (5 mmol), in CHCl_3_ by adding a freshly prepared aqueous solution of AgF (10 mmol) according to the procedure described in [22]. The reaction mixture was shaken vigorously for 20 minutes at room temperature. During this time, the colour of the organic phase changed from red to orange and finally to yellow. The yellow CHCl_3_ phase was separated from the aqueous phase, containing the white precipitate of AgCl, through a separatory funnel and filtered. Condensation of the yellow filtrate under reduced pressure gave a fluffy lemon-yellow solid that was dried *in vacuo* over silica gel and recrystallized from toluene. Yield 90% and m.p. 235°C (dec.) Lemon-yellow, needle-like crystals of **1** suitable for X-ray analysis were obtained by vapour diffusion of petroleum ether into a CHCl_3_ solution of the product placed in an H-shaped tube. Anal. Calc. for C_10_H_10_F_2_Ti (216.08): C, 55.59; H, 4.66. Found: C, 55.28; H, 4.62. Selected IR data (KBr, cm^−1^): 3108 (s), 1442 (s), 1362 (w), 1016 (s), 874 (m), 822 (vs), 610 (w), 564 (s), 539 (m). ^1^H NMR (400 MHz, CDCl_3_): *δ* 6.53 (t).

### 2.1. X-Ray Crystallographic Studies

A yellow prismatic crystal of **1** was taken directly from the mother liquid and immediately cooled to −113°C. Diffraction measurements were made on a Rigaku R-AXIS SPIDER Image Plate diffractometer using graphite monochromated Cu K*α* radiation. Data collection (*ω*-scans) and processing (cell refinement, data reduction and empirical absorption correction) were performed using the CRYSTALCLEAR program package [23]. Important crystal data and parameters for data collection and refinement are listed in Table 1. The structure was solved by direct methods using SHELXS-97 [24] and refined by full-matrix least-squares techniques on *F* ^2^ with SHELXL-97 [25]. Hydrogen atoms of the cyclopentadienyl (Cp) group were introduced at calculated positions as riding on bonded atoms. All non-H atoms were refined anisotropically. In the structure of **1**, the carbon atoms of one of the two Cp rings are disordered over symmetry-related positions. CCDC 771089 contains the supplementary crystallographic data for this paper. These data can be obtained free of charge at http://www.ccdc.cam.ac.uk/conts/retrieving.hmtl (or from the Cambridge Crystallographic Data Centre, 12 Union Road, Cambridge CB2 1EZ, UK; Fax: (internat.) ++ 44-1223/336-033; E-mail: deposit@ccdc.cam.ac.uk).

## 3. Results and Discussion

### 3.1. Synthetic Comments

The title compound was first prepared by Wilkinson and Birmingham in 1954 by dissolving the bromo analog, [Ti(*η* ^5^-C_5_H_5_)_2_Br_2_], in hot 12 *N *hydrofluoric acid and heating on a steam-bath until the solution was pale yellow in colour. On cooling, yellow crystals were received which were recrystallized from 3 *N* hydrofluoric acid solution [21]. Among the other published in the literature methods, we selected that described by Pink in his Thesis. The method uses AgF prepared *in situ*, which gives the best yields in the shortest time [29]. The preparation of **1 **involves the reactions represented by the stoichiometric equations (1)–(3):

(1) $$\begin{array}{cc} & 2{\text{AgNO}}_{3}+2\text{NaOH}\to {\text{Ag}}_{2}\text{O}+2{\text{NaNO}}_{3}+{\text{H}}_{2}\text{O}, \end{array}$$

(2) $$\begin{array}{cc} & \;\;\;{\text{Ag}}_{2}\text{O}+2\text{HF}\to 2\text{AgF}+{\text{H}}_{2}\text{O}, \end{array}$$

(3) $$\begin{array}{cc} & \left[\text{Ti}{\left(\eta^{5}\text{-}{\text{C}}_{5}{\text{H}}_{5}\right)}_{2}{\text{Cl}}_{2}\right]+2\text{AgF}\\ & \;\;\;\;\;\;\;\overset{{\text{CHCl}}_{3}/{\text{H}}_{2}\text{O}}{\to }\left[\text{Ti}{\left(\eta^{5}\text{-}{\text{C}}_{5}{\text{H}}_{5}\right)}_{2}{\text{F}}_{2}\right]+2\text{AgCl}. \end{array}$$

The two first steps ((1) and (2)) are necessary because AgF decomposes upon staying. Experiments with commercial AgF always lead to poor yields. Another method for a high-yield synthesis of **1** was developed by Ruzicka and coworkers [30] and involves the 1  :  2 reaction of [Ti(*η* ^5^-C_5_H_5_)_2_Cl_2_] and the good fluorinating agent {2-[(CH_3_)_2_NCH_2_]C_6_H_4_}(n-Bu)_2_SnF in CH_2_Cl_2_. 

Complex **1** is, like [Ti(*η* ^5^-C_5_H_5_)_2_Cl_2_], a very stable compound. It dissolves easily in common organic solvents such as chloroform, methanol, benzene, toluene and is much more soluble in water than are the other congener halides, even at room temperature, without decomposition. A decent water solubility is essential for a satisfactory cytotoxic activity of titanium(IV) complexes. Its molar conductivity (H_2_O, 10^−3^ M, 25°C) is less than 5 S cm^2^ mol^−1^. This means that, in contrast to the other corresponding titanocene dihalides, the fluoride ligands in **1** are hydrolytically stable. In this case, the water solubility and negligible molar conductivity could be attributed to the formation of hydrogen bonds between the electronegative F^−^ ligands of **1** and the H_2_O molecules rather than to a dissociation of the complex to species like [Ti(*η* ^5^-C_5_H_5_)_2_(H_2_O)_2_]^2+^ and F^−^. The oxophilicity of Ti^IV^ makes complexes of this metal ion with organic and inorganic ligands highly susceptible to hydrolysis. Complexes of hydrolytic instability are not biologically active, probably due to rapid formation of inactive aggregates [14].

### 3.2. Spectroscopic Characterization

The IR spectra of **π**-bonded cyclopentadienyl metal complexes have been studied [31–33]. In the IR spectrum of **1** the bands at 3108, 1442, 1016, 874/822 and 610 cm^−1^ can be assigned [31–33] to the **ν**(CH), **ν**(CC), **δ**(CH), **π**(CH) and *δ*(CCC) vibrational modes, respectively. The bands at 564 and 539 cm^−1^ are assigned [32] to the *ν*_1_(*A*_1_) and *ν*_6_(*B*_1_) (under *C*_2*V*_ point group symmetry) stretching modes of the terminal Ti^IV^-F bonds.

The ^1^H NMR spectrum (CDCl_3_) of **1** shows a triplet peak at *δ* = 6.53 ppm corresponding to the equivalent protons of the *η* ^5^-C_5_H_5_ protons [31, 34]. The triplet character of the signal is due to the small ^3^ *J* coupling (1.7 Hz) between the cyclopentadienyl protons and the fluoro nuclei in the molecule [34].

### 3.3. Description of Structure

**Figure 1:**
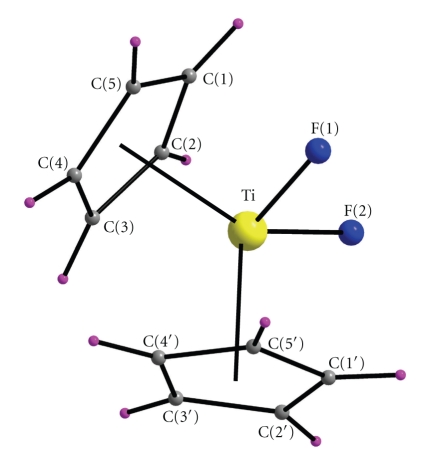
Labeled PovRay representation of complex **1** with the atom numbering scheme. Primes are used for symmetry-related atoms. Colour scheme: Ti^IV^, yellow; F, blue; C, gray; H, purple.

**Figure 2:**
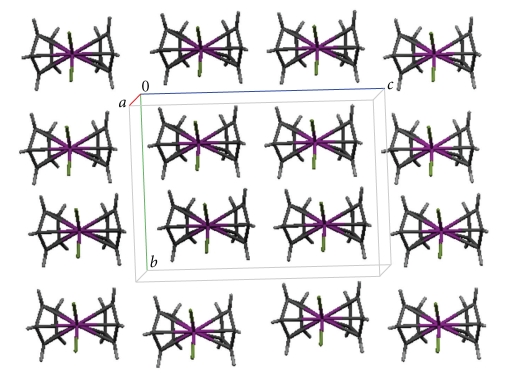
Drawing of the crystalline packing of complex **1**.

The molecular structure and a crystal packing diagram of **1** are shown in Figures 1 and 2, respectively. Bond lengths and angles are listed in Table 2.

The structure of **1** consists of isolated [Ti^IV^(*η* ^5^-C_5_H_5_)_2_F_2_] molecules. The molecule has the familiar, distorted tetrahedral shape found in the chloro [26], bromo [27] and iodo [28] members of the [Ti^IV^(*η* ^5^-C_5_H_5_)_2_X_2_] family of complexes. The distorted tetrahedral structure arises if we consider the Cp ring centroids as each occupying one coordination site around the metal ion. The cyclopentadienyl centroid-titanium-cyclopentadienyl centroid angle is 128.53°, and the fluorine-titanium-fluorine angle is 96.0°. The TiF_2_ group defines a symmetry plane and there is thus one crystallographically independent Cp ligand. The plane defined by the Ti^IV^ atom and the centroids of the Cp rings bisects the F-Ti-F bond angle. Thus, the molecule has a 2-fold symmetry about the line of intersection of this plane and the plane of the F-Ti-F bond angle; its point group symmetry is *C*_2v_. The Cp ring is planar to ±0.019 Å. The least-squares planes of the two symmetry related Cp rings in the bent (Cp)_2_Ti fragment of **1** form a dihedral angle of 53.49°. The two Cp rings exhibit a staggered conformation. The angle between the normals to ring planes is 53.83°.

The five Ti-C bond lengths range from 2.363(9) to 2.400(11) Å. This narrow range establishes a distinct *pentahapto* coordination mode for each Cp ligand in **1**. The mean Ti-C bond distance (2.380 Å) is in agreement with the corresponding values of other bis(cyclopentadienyl)titanium(IV) complexes, for example, the value of 2.370 Å in [Ti(*η* ^5^-C_5_H_5_)_2_Cl_2_] [26]. The Ti^IV^-F bond distances [1.853(4), 1.859(4) Å] are similar to those found in other 4-coordinate titanium(IV) complexes containing terminal Ti-F bonds [16, 35, 36]. The C-C bond lengths in the Cp ring of **1** with an average value of 1.388 Å are within the usual range reported for organometallic complexes containing Cp^−^ rings [27].

Since the single-crystal, X-ray structures of all the four members of the [Ti(*η* ^5^-C_5_H_5_)_2_X_2_] (X = F, Cl, Br, I) family are now known, we feel it is interesting to compare some of their important structural and crystallographic parameters. The comparisons are presented in Tables 3 and 4, respectively. The average distance from the Ti^IV^ atom to the ring centroid and the angle between the vectors from the metal center to each of the ring centroids vary by less than 0.025 Å and 4°, respectively, across the four complexes. The F-Ti-F angle (96.0°) is slightly wider than the corresponding X-Ti-X (X = Cl, Br, I) angles (92.8–94.5°) suggesting that electronic, rather than steric, effects influence this angle. The fluoro complex **1** has the largest X-Ti-X and the smallest Cp-Ti-Cp angle. As expected, the average titanium-halogen distances follow the sequence Ti-F < Ti-Cl < Ti-Br < Ti-I. 

## 4. Conclusions

The important message of this work is that we have structurally characterized the last member, namely the fluoro complex, of the [Ti(*η* ^5^-C_5_H_5_)_2_X_2_] (X = halogenide) family of complexes. Although the preparation of **1** was reported ~55 years ago, its exact molecular and crystal structure remained unknown until our report in this work. Studies are now underway in our laboratories to investigate the reactivity pattern of **1** with bidentate and tridentate N- or/and O-based ligands, and to study the hydrolytic behavior and cytotoxic activities of the resulting products. It should be mentioned that the fluoride ions themselves, if present in the products, are not cytotoxic at concentrations below 10^−3^ M [16].

## Figures and Tables

**Table 1:** Crystallographic data and structure refinement for complex **1**.

Empirical formula	C_40_H_40_Ti_4_F_8_
Formula weight (g mol^−1^)	864.32
Colour and habit	Yellow prisms
Crystal size (mm)	0.75 × 0.10 × 0.10
Crystal system	Orthorhombic
Space group	*C*2*cm *
*Unit cell dimensions*	
*a*, Å	5.9055(4)
*b*, Å	10.3021(5)
*c*, Å	14.2616(9)
**α**,°	90
**β**,°	90
**γ**,°	90
*V*, Å^3^	867.66(9)
*Z*	1
**ρ**, Mg m^−3^	1.654
*T*, K	160(2)
Radiation (Å)	Cu K*α* (**λ** = 1.54178)
**μ**, mm^−1^	8.207
*F*(000)	440
**θ** range (°)	8.61–59.99
*Index ranges*, °	–6 ≤ *h * ≤ 6
	–11 ≤ *k * ≤ 11
	–14 ≤ *l * ≤ 12
Measured reflections	2894
Unique reflections	613 (*R*_int _ = 0.0947)
Reflections used [*I *> 2*σ*(*I*)]	535
Parameters refined	60
GoF (on *F * ^2^)	1.100
*R*1^a^ [*I * > 2*σ*(*I*)]	0.0668
*w * *R*2^b^ [*I * > 2*σ*(*I*)]	0.1469
(Δ*ρ*)_max _/(Δ*ρ*)_min _, e Å^−3^	1.183/−0.680

^a^ *R*1 = Σ(|*F*_*o*_| − |*F*_*c*_|)/Σ(|*F*_*o*_|). ^b^ *w* *R*2 = {Σ[*w*[(*F*_*o*_^2^−  *F*_2_^2^)^2^]/Σ[*w*(*F*_*o*_^2^)^2^]}^1/2^.

**Table 2:** Selected bond lengths (Å) and angles (°) for complex **1**.^a^

Ti-F(1)	1.853(4)	Ti-C(3)	2.363(9)
Ti-F(2)	1.859(4)	Ti-C(4)	2.365(10)
Ti-C(1)	2.375(11)	Ti-C(5)	2.396(11)
Ti-C(2)	2.400(11)	Ti-Cp	2.066
C(1)-C(2)	1.358(15)	C(4)-C(5)	1.386(14)
C(2)-C(3)	1.396(14)	C(5)-C(1)	1.390(15)
C(3)-C(4)	1.411(16)	mean C-C	1.388

F(1)-Ti-F(2)	96.0(2)	F(1)-Ti-Cp	107.30
Cp-Ti-Cp′	128.53	F(2)-Ti-Cp	106.46
C(1)-C(2)-C(3)	108.6(10)	C(4)-C(5)-C(1)	107.5(10)
C(2)-C(3)-C(4)	106.8(7)	C(5)-C(1)-C(2)	109.2(13)
C(3)-C(4)-C(5)	107.8(8)	Aver. C-C-C	108.0

^a^Primed atoms are related to the unprimed ones by the symmetry operation *x*, *y*, 1/2-*z*.

**Table 3:** Comparison of some important molecular parameters (average values) for the four prototype titanocene dihalogenides, [Ti(*η* ^5^-C_5_H_5_)_2_X_2_].

Compound	Ti–Cp^a^ (Å)	Ti–X (Å)	Cp–Ti–Cp (°)	X–Ti–X (°)	Ref.
[Ti(*η* ^5^-C_5_H_5_)_2_F_2_]	2.066	1.856	128.5	96.0	present work
[Ti(*η* ^5^-C_5_H_5_)_2_Cl_2_]	2.059	2.364	131.0	94.5	[26]
[Ti(*η* ^5^-C_5_H_5_)_2_Br_2_]	2.058	2.493	131.6	94.9	[27]
[Ti(*η* ^5^-C_5_H_5_)_2_I_2_]	2.045	2.769	132.3	92.8	[28]

^a^Cp = *η* ^5^-cyclopentadienyl ring.

**Table 4:** Comparison of unit cell parameters for the four prototype titanocene dihalogenides, [Ti(*η* ^5^-C_5_H_5_)_2_X_2_].

Compound	*a* (Å)	*b* (Å)	*c* (Å)	**α** (°)	**β** (°)	**γ** (°)	Crystal system	Ref.
[Ti(*η* ^5^-C_5_H_5_)_2_F_2_]	5.9055(4)	10.3021(5)	14.2616(9)	90	90	90	Orthorhombic	present work
[Ti(*η* ^5^-C_5_H_5_)_2_Cl_2_]	7.882(5)	19.478(10)	12.156(9)	90.46(2)	102.58(2)	143.49(2)	Triclinic	[26]
[Ti(*η* ^5^-C_5_H_5_)_2_Br_2_]	7.872(5)	11.807(5)	12.310(3)	107.62(3)	100.83(4)	90.69(4)	Triclinic	[27]
[Ti(*η* ^5^-C_5_H_5_)_2_I_2_]	13.426(4)	7.173(2)	13.096(4)	90	116.686(5)	90	Monoclinic	[28]

^a^Cp = *η* ^5^-cyclopentadienyl ring.
